# Virtual manipulation of tail postures of a gliding barn owl (*Tyto alba*) demonstrates drag minimization when gliding

**DOI:** 10.1098/rsif.2021.0710

**Published:** 2022-02-09

**Authors:** Jialei Song, Jorn A. Cheney, Richard J. Bomphrey, James R. Usherwood

**Affiliations:** ^1^ School of Mechanical Engineering, Dongguan University of Technology, Dongguan, Guangdong, People's Republic of China; ^2^ Structure and Motion Laboratory, Royal Veterinary College, North Mymms, Hatfield, UK

**Keywords:** tail function, drag reduction, efficiency, bird flight

## Abstract

Aerodynamic functions of the avian tail have been studied previously using observations of bird flight, physical models in wind tunnels, theoretical modelling and flow visualization. However, none of these approaches has provided rigorous, quantitative evidence concerning tail functions because (i) appropriate manipulation and controls cannot be achieved using live animals and (ii) the aerodynamic interplay between the wings and body challenges reductive theoretical or physical modelling approaches. Here, we have developed a comprehensive analytical drag model, calibrated by high-fidelity computational fluid dynamics (CFD), and used it to investigate the aerodynamic action of the tail by virtually manipulating its posture. The bird geometry used for CFD was reconstructed previously using stereo-photogrammetry of a freely gliding barn owl (*Tyto alba*) and we validated the CFD simulations against wake measurements. Using this CFD-calibrated drag model, we predicted the drag production for 16 gliding flights with a range of tail postures. These observed postures are set in the context of a wider parameter sweep of theoretical postures, where the tail spread and elevation angles were manipulated independently. The observed postures of our gliding bird corresponded to near minimal total drag.

## 1. Introduction

Avian tails have many aerodynamic functions during steady gliding. Tails are used to maintain stability and balance as well as control turning [1–3]. When gliding, at slower speeds, tails spread and pitch downward, producing lift, which supplements that from the wings to support body weight and can also adjust the pitching moment [4–7]. Tails can also reduce drag and increase glide efficiency [8–12] in multiple ways. First, tails might alter the flow over the body and prevent flow separation, effectively streamlining the bird, which reduces body drag [8]. Second, the lift produced by the tail can alter the flow over the wings, modifying lift-induced (inviscid) drag [9]. Finally, the lift provided by the tail may allow the wing to operate at more efficient angles of attack, improving viscous efficiency [10]. Here, we examine whether the correlation between tail spread and pitch with glide speed is consistent with enhancing efficient gliding through modulating this wide array of drag-reducing mechanisms.

Owing to strong interactions between birds' wings, torso and tail, and the difficulties of controlling posture in flight experimentally, direct study of the aerodynamic functions of avian tails is challenging using cursory theoretical models or quantitative experimental measurement. High-fidelity computational fluid dynamics (CFD) modelling provides a new approach for studying the aerodynamic functions of tails because it can determine the detailed aerodynamic implications of a variety of virtually manipulated tail postures. By testing observed configurations adopted by birds and also sweeping through a range of virtual configurations that we do not observe, we can contextualize the natural geometries and identify where they appear on an optimality landscape. Critically, just as in real gliding birds, these different geometries do more than just modify drag; here, we account for the lift generated by these varied configurations by effectively adjusting the angle of attack of the whole geometry to maintain body weight support. This allows us to determine if added lift by the tail can allow the wings to operate at more efficient angles of attack and reduce overall drag. To predict the drag at the same body weight, or for any other like-for-like comparison, we introduce a tractable model for drag as a function of lift and parameterize it with the high-fidelity CFD simulations.

Our high-fidelity CFD is based on the detailed geometry of a barn owl (*Tyto alba*) acquired using multi-camera photogrammetry during steady gliding flight [13]. We validated the reference geometry, the tail of which is morphed, by comparing (i) qualitatively the vortex structures in the wake, and quantitatively comparing weight support, and (ii) the spanwise downwash against measured quantities using particle-tracking velocimetry (PTV). We also assess the effects of our computationally efficient Reynolds-averaged simulations against higher fidelity, but costly, large eddy simulations (LESs) for a subset of the morphed configurations.

With the drag model and validated simulations, we calculated the best aerodynamic performance (defined as minimum drag) achieved while producing weight support for a range of 42 different tail postures with varied spread and pitch. These 42 postures provide sufficiently dense sampling for us to interpolate among the postures and estimate the tail-posture optimality landscape for minimum drag. Finally, we compared the observed tail postures across 16 flights against our predicted postures based solely on drag minimization.

### 1.1. Drag model

**Figure 1.**
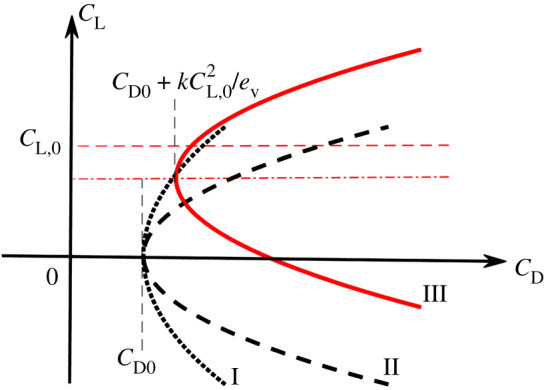
Comparison of drag models. Model I (dotted line) assumes *C*_pro_ and *C*_par_ to be constant, with a dependency on lift entirely due to induced drag. Model II (bold dashed line) assumes no camber effect. Our model (III, solid red line) includes the camber effect. The vertical grey dashed lines emphasize the minimum-drag coefficients achieved per model. Horizontal red lines indicate the lift coefficient due to the camber effect, *C_L,_*_0_ (dashed), and the line of symmetry for model III (dashed-dotted).

Drag is a critical parameter to understand because it has a direct effect on flight performance, the cost of flight and therefore a bird's ecology [14]. Although a variety of drag models have been proposed for bird flight [6,15–21], none is both convenient and sufficiently accurate to capture the effect of the tail manipulation, because of over-simplification of either the profile drag or parasite drag or the effect of camber. Since a reliable drag model is required to estimate the drag at constant body weight support, we modified a commonly used drag model [10,22] to include the effect of camber. The foundational model is a phenomenological description of the relationship between drag and lift. Figure 1 illustrates the comparison of our new model (model III) and previous models of varying simplification (models I and II). Our model is still simple, but has additional terms that have been ignored previously. To explain our model, we will contrast it with two models of reduced complexity.

Model I treats profile and parasite drag coefficients as constants [21] and behaves as

1.1 $$D=\left(\;\frac{C_{L}^{2}}{\pi AR\;e_{i}}+C_{D,\mathrm{pro}}+C_{D,\mathrm{par}}\;\right)qS,$$

where *AR* is the aspect ratio, *C*_D__,pro_ and *C*_D__,par_ denote the profile and parasite drag coefficients, respectively, *q* is the dynamic pressure, *S* is the planform area and *e_i_* is span efficiency. Span efficiency represents the effectiveness of producing lift: it is the lift-induced inviscid drag relative to the lowest achievable (mathematical definition in Material and methods). In this model, lift affects drag because it negatively modifies the flow over adjacent regions of the bird. It results in a gradual increase in drag with lift coefficient, with the increase due to the inviscid, induced drag component (figure 1). Drag model II [22] adds the quadratic rise in viscous, profile drag with lift (model II, figure 1), which behaves as

1.2 $$D=\left(\;\frac{C_{L}^{2}}{\pi AR\;e_{i}}+\frac{k_{C}C_{L}^{2}}{e_{v}}+C_{D,0}\;\right)qS,$$

where *k_C_* is an empirically derived curvature describing lift-to-drag performance, and the viscous efficiency coefficient

1.3 $$e_{v\;}=\frac{C_{L}^{2}}{\bar{C_{l}^{2}}}$$

describes the increase in drag due to inefficient loading across the span: for a wing of uniform sectional shape this is a departure from a constant, sectional-lift-coefficient distribution across the span, and *C*_L_ and *C*_l_ describe the lift coefficients for a whole wing and two-dimensional wing section, respectively. Model II provides an approximation that is valid for wing sections with negligible chord-wise camber—those for which the minimum drag occurs at zero lift; however, avian wings are usually highly cambered [23–26]. The barn owl wing geometry in this study reaches 7.4% normalized camber; in comparison, the middle wing section of a Boeing 737 is just 1.5%. Since a reliable drag model is required to estimate drag at constant body weight support, we modified the drag model (II) to account for the effect of camber. Our new model introduces an additional parameter, *C*_L,0_, the effect of camber on the lift coefficient, and drag now behaves as

1.4 $$D=\left(\frac{C_{L}^{2}}{\pi AR\;e_{i}}+\frac{k_{C}{\left(C_{L}-C_{L,0}\right)}^{2}}{e_{v}}+C_{D,0}\right)qS\;.$$

This model (model III, figure 1) offsets the minimum-drag condition to a point where positive lift is produced [22]. The quadratic relationship between lift and drag fits published avian data over the relevant range for this study (−5° to 7° for a different barn owl gliding configuration (*r*^2^ = 0.956) [26] and up to 13° on an extended hawk wing [7]).

### 1.2. Computational fluid dynamics modelling and its robustness

**Figure 2.**
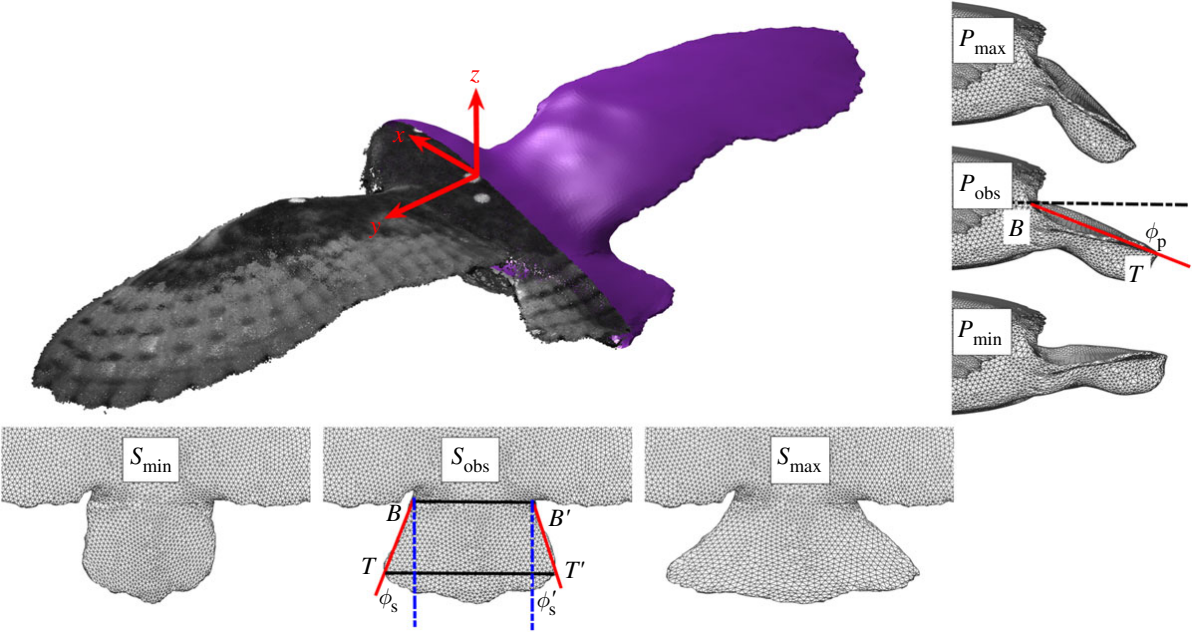
Point cloud obtained from stereo-photogrammetry and the geometry used for CFD simulations. The tail spread and pitch could be manipulated based on four landmarks. *B* and *B*′ determine the tail base and are defined as the points that give a minimal length when seen in vertical projection. Conversely, the tail tips *T* and *T*′ are defined by a connecting line that gives a maximal length when seen in vertical projection. The tail side edges are *BT* and *B*′*T*′. The projection of the angle between the side edges and glide trajectory on the sagittal plane gives the tail pitch angle, *ϕ****_p_***. The projection of the side edges when viewed from above gives the tail spread angle, *ϕ****_s_***. As the real tail is not perfectly symmetrical, we use the average of the two side edge angles to calculate both the spread and pitch angles. *S*_obs_ shows the observed tail spread angle (*ϕ****_s_*** = 17.5°), while *S*_min_ and S_max_ show the manipulated minimal and maximal spread, 0° and 41°, respectively. *P*_obs_ shows observed tail pitch angle (*ϕ****_p_*** = 26.0°), while *P*_min_ and *P*_max_ show the virtually manipulated minimal and maximal pitch, 6° and 46°, respectively.

The high-fidelity bird surface mesh for CFD is based upon the geometry of a trained adult female barn owl, *T. alba* (figure 2). The owl's geometry was obtained by stereo-photogrammetry [13]. In the specific flight from which the geometry was measured, the bird flew at a speed of *U* = 7.88 m s^−1^ with a glide angle of *θ* = 2.9°, which was close to the average for all 16 flights (*U* = 7.8 ± 0.4 m s^−1^ and *θ* = 3.4° ± 1.0°; mean ± s.d.) [13]. Further bird model geometry information is shown in table 1.

**Table 1.** Parameters of the barn owl, *Tyto alba*.

parameter	value
body mass, *m*	340 g
wing span, *b*	0.87 m
planform area, *S*	0.1478 m^2^
frontal area, *S_b_*	0.0095 m^2^
average chord length,$\;\bar{c}$	0.17 m
aspect ratio, *AR*	5.12
flight speed, *U*	7.88 m s^−1^
gliding angle, *θ*	2.9°

The smooth mesh vertices represented the point cloud closely, 96% mesh vertices were within 3 mm of the point cloud and 90% were within 2 mm (electronic supplementary material, figure S1; video S1 shows the error distribution on the geometry's surface). Smoothing was necessary as the original geometry retained surface-level irregularities, not representative of the actual bird. An additional 15 steady glides were measured for subsequent tail-posture comparison against our computed tail-posture optimality landscape for minimum drag.

We virtually manipulated the geometry's tail and systematically changed the spread angle, *ϕ_s_*, and pitch angle, *ϕ_p_*. The schematic in figure 2 shows the definition of these angles, highlighting the observed bird geometry and the limits of the manipulated postures in our parameter sweep. For the observed case, the tail spread angle was *ϕ_s_* = 17.5° and the tail pitch angle was *ϕ_p_* = 26.0°. We independently simulated tail spread angles from 0° ≤ *ϕ_s_* ≤ 41° with six intervals and tail pitch angles from 6° ≤ *ϕ_p_* ≤ 46° with seven intervals (see electronic supplementary material, video S2). These ranges comfortably envelop our observations of the gliding owl. We also simulated combinations of these parameters to test for interactions at the same gliding angle, giving a total of 42 tail postures, over which planform area ranges from 0.1444 to 0.1534 m^2^.

The flow around the gliding barn owl was simulated using commercial CFD software FLUENT 19.2 (ANSYS, Inc., Canonsburg, PA, USA) that solves the incompressible viscous governing equations of the fluid. The flow regime for this bird is expected to be turbulent, with Reynolds number approximately 88 000 using the mean wing chord ($\bar{c}=\;0.17\;m$) as our characteristic length. In order to simulate the flow accurately with a reasonable grid resolution, we used the *k* − *ω* SST turbulence model, which combines the advantages of the standard *k* − *ω* model and standard *k* − *ɛ* model, to give a satisfactory predictor of separated flows and adverse pressure gradient [27–30].

## 2. Results

### 2.1. Computational fluid dynamics validation and comparison to large eddy simulations

**Figure 3.**
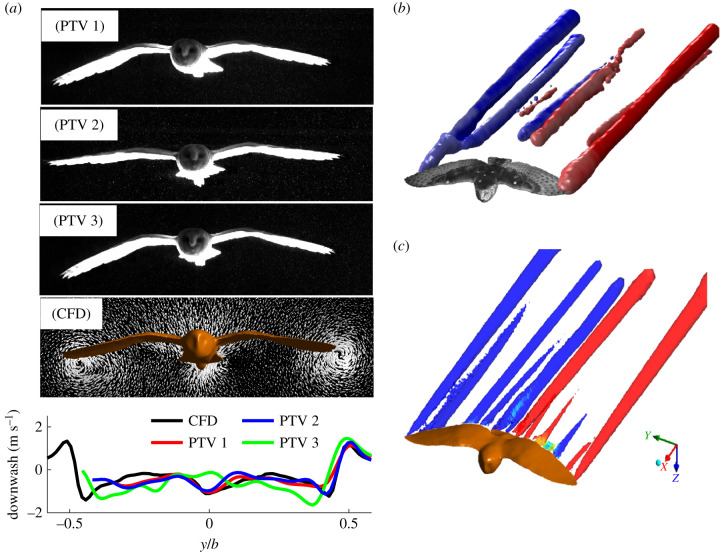
The comparison of simulated and measured downwash and vortices for a gliding *T. alba*. (*a*) Images of the posture adopted in each flight, along with the posture of the CFD surface model and the corresponding measured and computed downwash in the transverse plane. (*b*) The vortices in the wake measured using PTV (*Q*-value = 35 s^−2^; flight ‘PTV 1’) produce a complex wake pattern similar to that simulated (*c*). The downwash (*a*) in the CFD model (red) not only matches the measured magnitude across the wings but also shares a similar distribution of downwash across the normalized span. As the tail serves a number of functions during gliding flight, not surprisingly, downwash behind it varies slightly between flights.

At high Reynolds number, turbulence models are often introduced to efficiently simulate highly turbulent flow. However, there is no turbulence model specifically designed for the Reynolds numbers and geometries of flying birds. Therefore, we experimentally validated the observed (and centrally located) tail configuration in our CFD simulations. To achieve this, we measured the wake behind the same gliding barn owl using large-volume PTV, which tracked over 20 000 neutrally buoyant soap bubbles. We tested our CFD model by (i) comparing vertical force generation to weight support and (ii) by comparing the wake and downwash to those from the same individual gliding through tracked, neutrally buoyant soap bubbles (methods detailed in [11]). First, after accounting for the small vertical acceleration of the glide [13], simulated vertical force accounted for 94.2% of weight support. Second, the simulated downwash was in good agreement with the measured downwash both in magnitude and in distribution across the normalized span (figure 3).

The PTV provided confidence that the *k* − *ω* SST model could accurately reproduce the flow for the central tail configuration. To assess how error changed as we deviated from this configuration, we compared our lift and drag values as well as the span efficiency (*e_i_*) and viscous efficiency (*e_v_*) to those from higher fidelity LESs with the same fluid mesh (electronic supplementary material, figure S2). LES results for lift were systematically higher by 3.33 ± 1.04% (mean ± s.d.) than the *k* − *ω* SST model. Drag values were similar through the range of configurations studied, only underpredicting the sensitivity to tail pitch at higher angles of attack (with the drag difference ranging from −0.1% at small pitch *ϕ_p_* = 6° to 5.8% at the highest pitch angle *ϕ_p_* = 46°). Differences in *e_i_* and *e_v_* with changing tail pitch were similar: *e_i_* fluctuated and differed by at most 3.18% span efficiency, while our model underpredicted *e_v_* sensitivity to tail pitch, but the difference was at most 1.47% viscous efficiency. In general, the highest tail pitch had the greatest error, but the values were similar and overall trends for the four variables were the same.

### 2.2. Force production and flight efficiency of virtually manipulated tail postures

**Figure 4.**
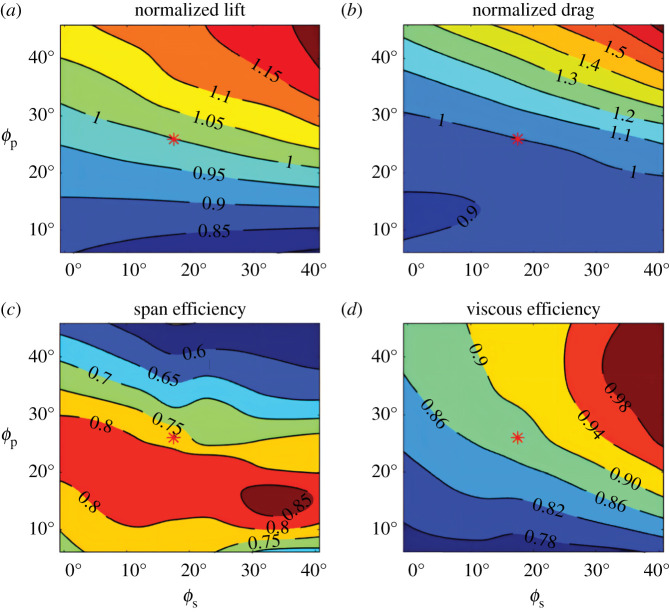
Forces and efficiencies across manipulations of tail spread and pitch. Normalized lift (*a*) and normalized drag (*b*) relative to the force of the observed tail posture, and overall span (*c*) and viscous (*d*) efficiency.

Despite the tail's role in enhancing lift, the force produced over the region of the tail is quite low. For the observed tail posture, the integrated pressure distribution over the tail area (3.5% of planform area *S*) accounts for just 3% of body weight. However, changes to the tail's spread and pitch dramatically alter the lift generation of the entire bird, ranging from 84% (*ϕ_s_* = 42°, *ϕ_p_* = 6°) to 122% (*ϕ_s_* = 42°, *ϕ_p_* = 46°) of body weight (figure 4*a*). Even though the lift produced by the tail alone is small, the tail alters the flow around the bird's body and the proximal sections of the wings, conferring substantial control authority. The tail therefore acts as an aileron, effectively changing the aerodynamics of the entire lifting surface (electronic supplementary material, video S3). With changes in tail spread and pitch, the total drag on the bird also changes (figure 4*b*) and ranges from 88% to 172% of the observed case. In our simulations, flow separation occurred in the sagittal plane when the tail was pitched at 46° (electronic supplementary material, figure S3), and flow attachment was likely to be enhanced by the strong tip vortices of the tail (electronic supplementary material, figure S4).

As tail posture changes, the span loading and downwash distribution behind the bird varies. Across the simulated tail postures, span efficiency, *e_i_*, ranges from 0.55 to 0.86 (figure 4*c*) and viscous efficiency, *e_v_*, ranges from 0.78 to 0.99 (figure 4*d*). Importantly, the regions of high efficiency are not aligned and require different degrees of tail pitch. High span efficiency occurs when the tail is widely spread with a moderate pitch angle (*ϕ_s_* = 36° and *ϕ_p_* = 15°), while high viscous efficiency occurs when the tail is widely spread and pitched down (depressed).

### 2.3. Adjusting the simulated drag for constant weight support

Steady gliding flight requires lift force to balance body weight. Changes to tail posture alone, without changing either flight speed or the orientation of the bird, only allows for postures that produce the same weight support to be compared like for like (figure 4*a*; e.g. the isocline for normalized lift = 1). To compare postures that produce deficit or surplus lift, we must account for the effect of that lift on our measurements. As drag depends upon the lift quadratically within a moderate domain of angles of attack, small deviations in lift can have large effects on drag. We account for deviations in lift by effectively modifying the pitching orientation of the bird and therefore angle of attack of the whole geometry. The drag model allows us to modify the simulated drag values, increasing drag when lift is in deficit, and decreasing drag when lift is in surplus. Here, that range is 16% deficit to 22% surplus (figure 4*a*).

**Figure 5.**
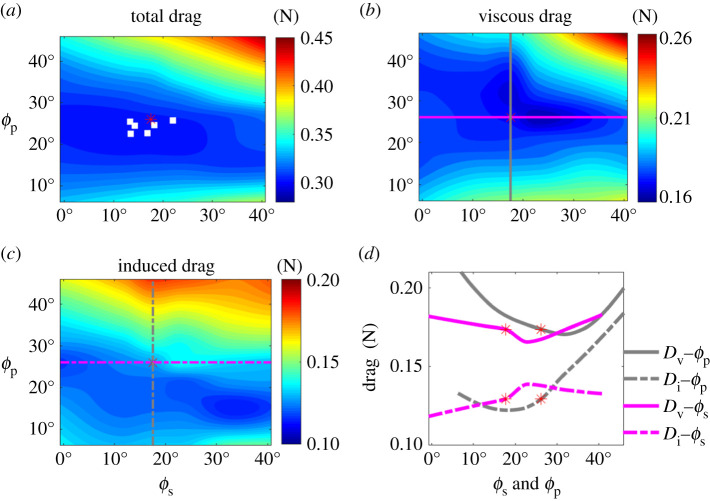
Drag predicted by the drag model with the same body weight support for different tail spread and pitch angles. (*a*) Total drag; white squares show the tail posture of observed flights within 2% of the speed of the observed tail-posture case used for simulation. Total drag is the sum of the (*b*) viscous drag and (*c*) induced drag. (*d*) Change in viscous (solid line) and inviscid (dashed line) drag as a function of pitch (grey) and spread (magenta) angle originating from the observed posture. Data are extracted in transects shown in (*b*) and (*c*).

To estimate how tail posture changes drag, while maintaining constant weight support, we use our new model, which included the effects of camber (III—equation (1.4)). With parameters *e_i_*, *e_v_*, *C*_L_*_,_*_0_ and *C*_D,0_ estimated in the drag model by CFD (see ‘Solving for aerodynamic model coefficients using CFD’ in Material and methods), the drag of all 42 tail postures was determined with the same body weight support (figure 5*a*). Minimum drag now occurs at spread angle *ϕ_s_* = 18° and pitch angle *ϕ_p_* = 23° when gliding at 7.88 m s^−1^. Of 16 measured glides of the same barn owl, six flights occur at speeds within 2% of 7.88 m s^−1^ and are located in the region of minimal total drag. We can delve further into this drag analysis. As described above, viscous drag is the remaining drag after subtracting induced drag from total drag. Minimal viscous drag is achieved at *ϕ_s_* = 23.2°, *ϕ_p_* = 25.6°, while minimal induced drag is achieved at *ϕ_s_* = 15.0°, *ϕ_p_* = 36.1°. At the measured tail posture, induced drag and viscous drag contribute 32.3% and 67.7% of the total drag, respectively (figure 5*b*,*c*), making viscous drag twice as important as inviscid drag for barn owls at these speeds.

Deviating from the observed tail posture resulted in viscous drag increasing more dramatically than induced drag when the tail pitches up, and the opposite is true when the tail pitches down (figure 5*d*). As minimal viscous drag and induced drag cannot be achieved simultaneously—i.e. with the same tail pitch and spread angles—the barn own appears to be minimizing total drag with a tail pitch angle compromising between the minimal viscous drag pitch angle (*ϕ_p_* = 35.8°) and the minimal induced drag pitch angle (*ϕ_p_* = 19.8°).

### 2.4. Variation in drag with flight speed

To provide confidence in the robustness of the result that the tail posture adopted minimizes drag, we compared tail-posture results against our predictions across a range of flight speeds, repeating our drag predictions for a range of tail pitch and spread angles at four additional speeds: 6.5, 7.47, 8.49 and 9.0 m s^−1^. The parameters *e_i_*, *e_v_*, *C*_L_*_,_*_0_ and *C*_D,0_ were treated as independent of flight speed, and *k* was treated as linearly related to the Reynolds number with *k* = −6.32 × 10^−7^*Re* + 0.166 (*N* = 20, *r*^2^ = 0.62) estimated from data collected by other research [24,25]. However, as we find below (sensitivity to *k*), drag's sensitivity to *k* over the range relevant to this study was low, allowing for variation beyond Reynolds number effects. This left *C*_L_ as the remaining variable.

**Figure 6.**
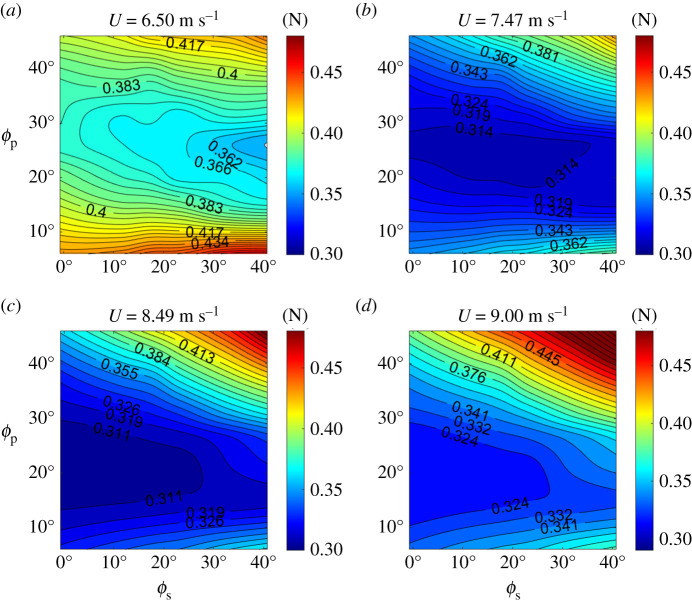
Total drag predicted by the drag model with different tail postures at four flight speeds (*U* = 6.50 m s^−1^, 7.47 m s^−1^, 8.49 m s^−1^ and 9.00 m s^−1^).

Generally, at the lowest speed (*U* = 6.50 m s^−1^) and highest speed (*U* = 9.00 m s^−1^), drag is greater than it is for the case of *U* = 7.88 m s^−1^ for all postures, indicating that the barn owl chose to glide at an optimal flight speed for minimal drag with that tail posture or, conversely, that tail posture was chosen to minimize drag at that speed (figure 6). At each flight speed, there is an alternative tail posture that has lower drag. However, the minimal drag for some speeds (e.g. *U* = 6.50, 8.49 and 9.00 m s^−1^) was beyond the range of tail spread and pitch angles in our parameter sweep. Interestingly, near the minimum-drag domain, drag becomes less sensitive to tail posture (indicated by the increased isocline spacing in figure 6). In these regions, the tail could be used for control with little additional cost in terms of flight efficiency.

**Figure 7.**
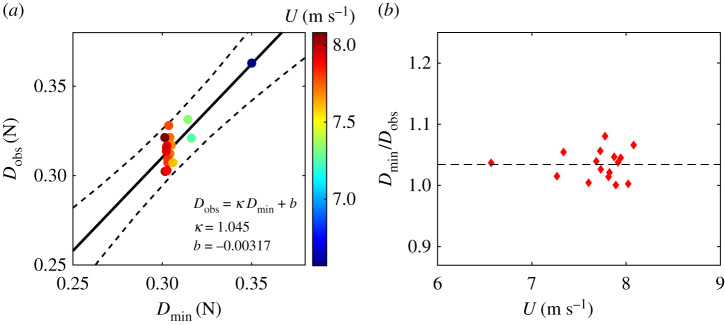
(*a*) The drag estimated with 16 observed tail postures versus the minimal drag prediction from the drag model. Circle colour denotes flight speed. The solid line denotes the linear fit of observed drag versus theoretical minimal drag using model *D*_obs_ = *κD*_min_ + *b* (*κ* = 1.043, *b* = −0.00265, *N* = 16, *r*^2^ = 0.760), and the dashed lines denote the 95% confidence interval. (*b*) The correlation between *D*_obs_/*D*_min_ and flight speed, *U* (*r*^2^ = 1.7 × 10^−4^).

The estimated drag for the 16 observed tail postures closely matched the predicted drag from our drag model (figure 7). The accuracy of the model did not depend upon flight speed (figure 7*b*; *r*^2^ = 1.7 × 10^−4^). While on average observed drag was approximately 3.5% greater than the predicted minimum (figure 7*a*; prediction offset *b* was −0.85% of minimal drag and prediction slope was 1.043% of minimal drag), the agreement between models supports the notion that the selected tail posture was operating near minimal drag across flight speeds.

## 3. Discussion

### 3.1. Aerodynamic roles of the tail

We have described how the tail operates to reduce total drag. Previous studies have shown that, at low speed, birds spread their tails widely and operate them at a high pitch angle relative to the flight direction, indicating that birds use the tail to generate lift in order to support body weight [12,21,31]. From our results, we can see how modulating tail posture instead of whole bird posture, or wing posture, can produce weight support with less overall drag (figure 5*a*). For a speed of *U* = 6.50 m s^−1^, the tail posture that supported body weight and produced the least drag was with a high spread angle and relatively large downward pitch angle, as observed in natural flight. Despite the tail's large effect on lift and drag, changes in tail posture disproportionately affect the wings and body (figure 4*a*; electronic supplementary material, video S3). Integrating the tail's surface pressure alone contributes just 3% of body weight support, a similar value to that measured in a flying starling (2%) [4] but less than for a pigeon (7.9%), measured using differential pressure sensors on the tail [32].

For a flying bird, a stable configuration might reduce energy consumption during long-distance flight by reducing control inputs [33]. The bird would become more stable longitudinally were the tail to pitch up and provide longitudinal dihedral, along with the wings sweeping back for trim. However, the negative angle of attack required of the tail would also reduce lift and introduce extra drag (figure 3*b*). This posture is not used by the barn owl but can be observed momentarily as pigeons prepare to land [32]. As the tail usually pitches up, a reverse cambered body–tail chord confers longitudinal stability, which suggests that the tail can also act as a pitch stabilizer.

### 3.2. Variation with speed

While minimizing drag and the energy cost associated with flight per distance—minimizing cost of transport—is a common glide strategy [21], birds also frequently employ a slightly costlier strategy that allows them to fly slower and minimize their sink speed [5]. We can evaluate the tail postures of the barn owl flights for these two strategies by computing the ‘glide polar’, the effect of flight speed on sink speed. As wind tunnel studies have shown that wing span and area for gliding animals decreases as flight speed increases [5–7,21], we allowed area to change as a function of flight speed too. As birds glide at small angles to the horizon, we can approximate the steady-state lift generated as *L* ≈ *W*, where *W* is body weight*,* and then substituting *C*_L_ *= L*/(*qS*) ≈ *W*/(*qS*) (where *q* = 0.5 *ρU*^2^) into equation (1.4) gives

**Figure 8.**
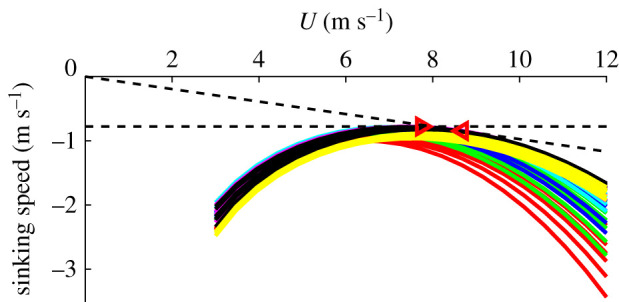
Gliding polar of a barn owl. Lines denote the polar for different tail postures; the lines with the same colour denote the case with the same tail pitch. The triangle on the left (vertex: (7.74, 0.778) m s^−1^) denotes the speed yielding the minimal sinking rate while the triangle on the right (vertex: (8.68, 0.846) m s^−1^) denotes the speed yielding the maximum gliding range.

3.1 $$D=\frac{W^{2}}{\pi AR\;e_{i}qS}+\frac{k_{C}W^{2}}{e_{v}qS}-2k_{C}/e_{v}\;C_{L,0}W+(k_{C}/e_{v}\;C_{L,0}^{2}+C_{D,0})\;qS,$$

which allowed us to compute overall drag as a function of speed and area. With this drag model, we can estimate the gliding polar of the bird (figure 8). This polar reveals the speed giving the minimum sink rate to be *U_ms_* = 7.74 m s^−1^, while the speed for maximum range (maximizing lift-to-drag ratio, or minimum drag for constant weight support) is 12% faster at *U_bg_* = 8.68 m s^−1^. As nine out of 16 observed flights were within 2% of *U_ms_*, we can conclude that the barn owl chose a speed that would minimize the sinking rate when gliding through the flight corridor.

### 3.3. A note on diversity, form and function of bird tails

Bird tails are immensely diverse in their form—ranging from the highly exaggerated peacock trains (tail coverts) to the nearly absent tails of adult bateleur (eagle). It is reasonable to suppose, then, that there may also be a diversity of functions, in terms of both signalling and aerodynamics. Among the aerodynamic roles, the focus of this paper has been on drag minimization. However, we certainly do not suggest this to be the primary selective pressure on tail form: presumably aspects of dynamic stability and control are hugely important. But what *is* proposed here is that, for a *given* planform and tail, the tail spread and incidence observed in gliding are consistent with drag minimization, and we introduce the aerodynamic phenomena behind this drag minimization. It is reasonable to suggest that species with very different tail morphologies might also prioritize drag-minimizing spread and posture during gliding. However, even though previous studies demonstrate tail function (variation in spread, Harris' hawk [1]) and lift production (in slow pigeons [32], swift [21,34], jackdaw [6]) consistent with the drag-minimizing principles described here, it is difficult to know how general drag minimization is at this stage.

The mechanism behind drag minimization described here—involving a compromise between viscous and inviscid drag minimization—contrasts with those proposed that focus on inviscid drag minimization [8,10,12,35], which fail to account for the sense and location of the observed and modelled trailing vortices [11]. The potential drag-reducing role of the tail as a splitter-plate controlling flow separation and vortex shedding [4] is intriguing; however, it is difficult to be certain that the counterfactual ‘without-tail’ case is suitable for comparison—*could* the body be streamlined without a tail? The study here limits exploration of counterfactual possibilities to those reasonably possible given simple geometric transformations of the observed tail. Exploring the implications of very much shorter or longer tails would be fascinating and worthy of future work.

### 3.4. Robustness of turbulence models

We employed a Reynolds-averaged Navier–Stokes (RANS) turbulence model, *k* − *ω* SST, to predict the fluid dynamics of the barn owl. For the observed flight posture, there is a qualitative similarity in the measured and simulated wakes and little difference between downward flow velocities beneath the bird. Additionally, the simulation predicts 94.2% body weight support. The agreements are striking and provide reassurance that the CFD is accurate, at least around the central configuration.

The degree to which we can deviate from this central configuration though is unclear. We cannot validate extreme tail configurations and estimate the scaling of the error in the *k* − *ω* SST model, as the bird chooses not to fly in these—presumably maladaptive—configurations. Indeed, the *k* − *ω* SST model might be impaired at large tail pitch angles, as its performance is less well suited to capturing flow separation [36]. However, the model is generally sufficient to predict the aerodynamics to an acceptable degree [27–29], especially when there is no expectation of systematic bias in a parametric study.

To address the robustness of the *k* − *ω* SST model for our tail postures with high pitch angle, we compared a subset of the lift and drag results to those from LESs using the same fluid mesh, which generally performs better for large angles of attack/pitch. LES results for lift and drag were similar to those from the *k* − *ω* model (electronic supplementary material, figure S2). LESs systematically estimated 3.33 ± 1.04% more lift than the *k* − *ω* model, while drag values too were similar, except at the most extreme tail pitch angle (mean: 1.92 ± 2.23%). The differences are sufficiently negligible to not affect the conclusions of this study. That the validated central tail configuration reproduced the flow around a live bird, and that our RANS model agrees with the higher fidelity LES model away from this central configuration, demonstrated that, for force measurement, the *k* − *ω* SST model is sufficient for our geometry and configuration domain.

### 3.5. Sensitivity to *k_C_*

In the two-dimensional drag model, the parameter *k* describes the quadratic rise in *C*_d_ with *C*_l_ – *C*_l,0_, and varies with Reynolds number. It has been suggested that the same *k_C_* can be used for both two-dimensional and three-dimensional wings [22], which means that the *k_C_* for any chord is constant; we adopted this assumption. The determination of *k_C_* is based on fitting the three-dimensional drag model to the CFD simulated *C*_L_–*C*_D_ polar at five angles of attack. Aside from obtaining *k_C_* from the observed tail case (*k_C_* = 0.110), we can compute alternative values of *k_C_* based on the equivalent data for the four extreme postures: *k_C_* = 0.0845, 0.0839, 0.0903 and 0.114, deviating by −23% to 4% from our observed case. Over this range of values, if we had selected an alternative *k_C_* from a configuration with higher uncertainty, the overall results would have been similar, as total drag is relatively insensitive to variation of *k_C_* in the model, when lift equals weight support. The change in drag coefficient with respect to the change in *k_C_* can be calculated as follows:

3.2 $$\frac{dC_{D}}{dk_{C}}=\frac{{\left(C_{L}-C_{L,0}\right)}^{2}}{e_{v}}∼\;0.014,$$

or, alternatively, we find an approximately 0.3% change in drag coefficient for every per cent change in *k_C_*.

### 3.5. Sources of error and limitations

With the increased accuracy of the LES model, the optimality landscape could change, but likely by a small amount. Importantly, differences in lift and drag between *k* − *ω* and LES models are small, as are the gradients in per cent error with respect to tail pitch angle (electronic supplementary material, figure S2). The systematic error in lift would have no effect on the landscape other than lowering the overall drag as a result of the *k* − *ω* model slightly underestimating lift. The difference in drag between the LES model and the *k* − *ω* model, while small, does increase with tail pitch angle. The gradient of this per cent error is nonlinear, and while greatest at the extreme tail pitch angle, it decreases as it approaches the region of minimum drag; the LES model would probably predict a steeper slope at high tail pitch angles, but would not shift the region of minimal drag substantially. Therefore, we expect that our conclusion that birds operate their tail to minimize drag is robust to the chosen CFD model.

A limitation of our drag model is that it assumes a quadratic relationship between lift and drag, which is an approximation that works best when induced drag is small and three-dimensional effects are weak [22]. Therefore, this is an imperfect approximation, but one that only serves to adjust the simulated conditions for body weight support slightly. Our drag model ignores nonlinear effects and interactions among the drag parameters (profile, parasite and inviscid), but these interaction effects are likely to be small, as we are generally only making small adjustments to lift, and therefore drag (the most extreme changes in lift are: maximum deficit 16% and surplus 22%); further, the linearized drag model fits well to lift–drag polars for the barn owl regardless of configuration (electronic supplementary material, figure S5). Most importantly, the drag value that is being adjusted is one from simulation that captures the complex interactions between the tail and wings; and for which the observed posture, central among all of the postures, captures downwash and weight support well, and reproduces the wake pattern.

## 4. Conclusion

In this paper, we developed a comprehensive analytical drag model, calibrated by high-fidelity CFD, to investigate the aerodynamic action of the tail by virtually manipulating its posture. Using this CFD-calibrated drag model, we predicted changes in drag when varying the tail pitch and spread angles while maintaining body weight support. By comparing the minimum predicted drag that was possible within a wide range of tail postures against the drag calculated for 16 observed gliding flights, we found that the observed postures of the gliding barn owl corresponded to near minimal total drag. This suggests that the barn owl adjusts its tail posture for minimal total drag and the mechanism by which it achieves that performance is by compromising between the ideal postures required to minimize the induced and viscous components of the total drag. Despite focusing here on the barn owl, flow visualizations around other birds (including the tawny owl, goshawk and swift) show similar characteristics: a strong downwash beneath the torso/tail when gliding [11,21]. Therefore, we believe that this is a common phenomenon for gliding birds. This result shows the mechanism by which birds' tails, which are most conspicuously used for manoeuvring or trim, can also be used to reduce the burden of drag, which runs against the common designs for bird-sized tailless air vehicles. The aeronautical motivation to design tailless aircraft is to reduce wave and induced drag [33,35,37,38]. In contrast with those large, fast-flying vehicles, small and slow vehicles on the scale of birds experience a different combination of drag components that can be reduced by manipulating the posture of a bird-like tail.

## 5. Material and methods

### 5.1. Geometry from stereo-photogrammetry and mesh reconstruction

The solid-body geometry of the barn owl in free flight was obtained from Cheney *et al*. [13]. The solid body was reconstructed from an indoor glide of a barn owl using photogrammetry and 12 high-speed cameras. Geometry manipulation was completed using SpaceClaim (Ansys Inc.) to smooth and gap fill the tail–body transition. Live-animal work was approved by the Ethics and Welfare Committee of the Royal Veterinary College (URN2018 1836-3) and methods which are further detailed in [11] and [13].

### 5.2. Computational fluid dynamics simulation

**Figure 9.**
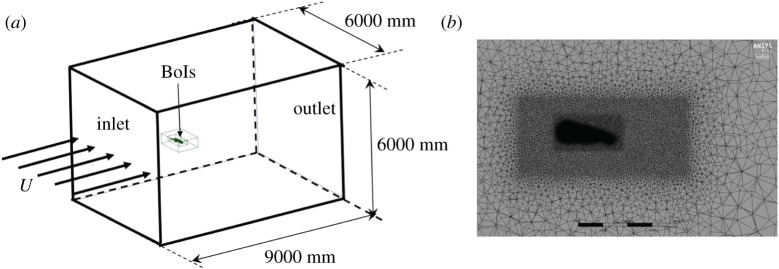
CFD simulation set-up (*a*) and the mesh on the sagittal plane of the computational domain (*b*). Each black scale bar in (*b*) denotes a length of 150 mm.

The simulation domain was 9000 × 6000 × 6000 mm^3^, and the bird model was placed 3000 mm downstream from the inlet (figure 9*a*). The fluid domain mesh was generated by ANSYS Mesh 19.2 (ANSYS, Inc., Canonsburg, PA, USA). We used a hybrid mesh, including tetrahedral, pentahedral and hexahedral cells with multiple bodies of influence (BoIs) to discretize the fluid domain (figure 9*b*). Over the surface of the bird, element size was less than 0.6 mm, with 283 elements across the average wing chord and 1450 elements across the span. Adjacent to the bird surface, the inflation layer had a first layer thickness of *δ_t_* = 0.1 mm (*y*+ = 3), a growth factor of 1.2 and 19 layers. Mesh independence is shown in table 2, justifying the use of *δ_t_* = 0.1 mm in this study. Two bodies of influence were used to control mesh size, with the mesh size in the inner domain being 5 mm and the outer domain being 12 mm. Overall, the number of elements was approximately 29 million, and the number of vertices was approximately 67 million.

**Table 2.** Mesh convergence study. The first layer thickness was changed while the inflation thickness was maintained. The use of *δ_t_* =0.1 mm gives 1.55% and 0.25% difference from the finest mesh (*δ_t_* = 0.08 mm).

first layer thickness	lift	drag
*δ_t_* = 0.08 mm (*y*+ ∼2)	3.22	0.2834
*δ_t_* = 0.1 mm (*y*+ ∼3)	3.17 (1.55%)	0.2827 (0.25%)
*δ_t_* = 0.2 mm (*y*+ ∼7)	3.15 (2.17%)	0.2925 (3.21%)

Commercial software FLUENT solved the fluid dynamics governing equations using a two-equation *k* − *ω* SST turbulence model. The velocity at the inlet was constrained to 7.88 m s^−1^ (*U*) (figure 9*a*), and at the outlet followed the Neumann boundary condition. Pressure at the inlet and outlet followed the Neumann boundary condition as well. The far-field boundary conditions, perpendicular to the flow, were constrained to be symmetric. Turbulence intensity was specified as 1% at the inlet to simulate the turbulence of flow in the flight corridor, which we expect to be greater than in a high-quality wind tunnel.

We considered the simulation solution to be converged when three unitless scaled residuals reached a minimum threshold: the continuity residual less than 5 × 10^−3^; each of the orthogonal velocity-component residuals less than 5 × 10^−7^; and the turbulence kinetic energy less than 3 × 10^−4^. In general, turbulence kinetic energy was the last criterion met. The residuals were scaled by the maximum residual value among the first five iterations of the simulation. The time step for each simulation was 0.1 ms.

### 5.3. Solving for aerodynamic model coefficients using computational fluid dynamics

We computed 62 fluid simulations around the bird model: 42 simulations were at the same angle of attack with systematically varying tail pitch and spread; the additional 20 simulations were to compute the aerodynamic polar for the reference tail-posture case, and four additional tail postures bounding the observed flight data (electronic supplementary material, figure S5).

The five unknown aerodynamic coefficients in our model are the viscous and inviscid span efficiency (*e_v_* and *e_i_*), empirically derived curvature of the polar (*k_C_*), minimum-drag coefficient (*C*_D,0_) and camber-induced-lift coefficient (*C*_L,0_). For all flights, we solve for viscous and span efficiency using the distributions of lift coefficients and downwash, respectively (equations (1.3) and (5.1)). We assume that polar curvature is negligibly different across tail morphology, and use the quantity derived from the reference tail-posture case, which is roughly centred in our pitch-spread parameter sweep. For the five cases with additional aerodynamic polar data, we solved for the minimum-drag coefficient and camber-induced-lift coefficient by fitting using the linear, least-squares method. We observed that, for each of these five cases, drag produced at the natural glide angle was roughly equal to the computed drag at zero lift; we do not believe that this has to be a general phenomenon, but use it here as a convenient model heuristic. This allows us to compute camber-induced lift and minimum drag, for flights with a single point on the aerodynamic polar, by adding the assumption that drag at zero lift equals drag at the natural glide angle.

### 5.4. Drag model calibration

**Figure 10.**
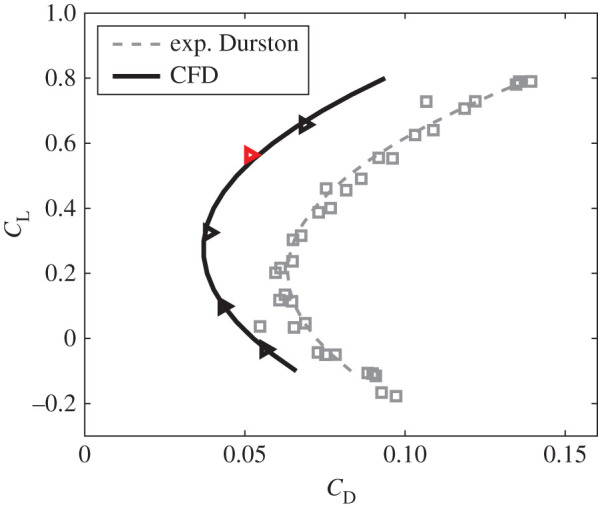
*C*_L_–*C*_D_ relationship for five angles of attack at 2.5° intervals. The red triangle denotes the barn owl with observed posture at its observed angle of attack of 2.9°. The *C*_L_–*C*_D_ is fitted with a binomial. Wind tunnel measured data from a three-dimensional-printed barn owl model in a different configuration is shown for comparison [25]. This model also shows the offset in lift due to camber.

To estimate the change in drag with constant weight support, we use drag model III, which includes the effect of camber (equation (1.4)). In model III, the coefficient *k* is an unknown parameter that can only be obtained accurately from the *C*_L_–*C*_D_ polar plot by fitting a quadratic function. By changing the angle of attack of the entire bird (with its observed tail posture), we obtained five *C*_L_ versus *C*_D_ points (figure 10). Fitting these points with a binomial gives *C*_D_ = 0.206 * *C*_L_^2^ – 0.113 * *C*_L_ + 0.0526 (*N* = 5, *r*^2^ = 0.980) for the three coefficients, respectively. Justification for this quadratic fit comes from experimental data also from a barn owl in a different configuration [26], which gave *C*_D_ = 0.215 * *C*_L_^2^ – 0.087 * *C*_L_ + 0.072 (*N* = 34, *r*^2^ = 0.956). The drag efficiency coefficients, *e*_*i*_ and *e_v_*, are related to the spanwise distribution of downwash and lift force, and are not sensitive to the angle of attack (electronic supplementary material, figure S6). Then, parameterizing equation (1.4) with the fitted function gives *k* = 0.110, *C*_L__,0_ = 0.452 and *C*_D,__0_ = 0.0270. It is impractical, owing to constraints on simulation time, to perform the same array of CFD simulations for all 42 tail postures, each of which would require simulations at a range of angles of attack. We simulated the range of angles of attack for just five tail postures to explore the sensitivity of the drag model to our assumptions (electronic supplementary material, figures S4 and S6).

Since *k_C_* is typically treated as a constant varying only with Reynolds number, its value for different tail postures is maintained at *k_C_* = 0.110 based on the polar from the barn owl's observed posture. From the *C*_L_–*C*_D_ polar in electronic supplementary material, figure S5, we observed that the minimum drag occurred when *C*_L_ was half the simulated value, approximately 0.2–0.3, and therefore owing to the function being parabolic, the drag at *C*_L_ = 0 was also equal to our simulated drag. That approximation was sufficient to derive the unknown parameters *C*_L__,0_ and *C*_D,__0_ (electronic supplementary material, figure S7). In general, increasing either spread or pitch increased both *C*_L__,0_ and *C*_D,__0_. The comparison between the *C*_D,__0_ using these assumptions and the CFD polar fit using the five simulated angles of attack gives, at most, 8.02% difference (electronic supplementary material, figure S8a) and the overall drag differed by 4.39% (electronic supplementary material, figure S8b).

### 5.5. Span efficiency *e_i_*

Span efficiency is calculated by the equation

5.1 $$e_{i}=\frac{L^{2}}{\pi \rho AR\;D_{\mathrm{ind}}}\;\mathrm{and}\;D_{\mathrm{ind}}=\int_{-b/2}^{b/2}\rho \Gamma (y)w(y)\;dy,$$

where *Γ*(*y*) and *w*(*y*) are the wing boundary circulation and downwash distribution along the span, respectively.

## Data accessibility

This article has no additional data.
